# Conductive Electrospun Nanofiber Mats

**DOI:** 10.3390/ma13010152

**Published:** 2019-12-31

**Authors:** Tomasz Blachowicz, Andrea Ehrmann

**Affiliations:** 1 Institute of Physics—Centre for Science and Education, Silesian University of Technology, 44-100 Gliwice, Poland; tomasz.blachowicz@polsl.pl; 2 Faculty of Engineering and Mathematics, Bielefeld University of Applied Sciences, 33619 Bielefeld, Germany

**Keywords:** electrospinning, conductive nanofibers, conductive solution, conductive polymers, conductive coating

## Abstract

Conductive nanofiber mats can be used in a broad variety of applications, such as electromagnetic shielding, sensors, multifunctional textile surfaces, organic photovoltaics, or biomedicine. While nanofibers or nanofiber from pure or blended polymers can in many cases unambiguously be prepared by electrospinning, creating conductive nanofibers is often more challenging. Integration of conductive nano-fillers often needs a calcination step to evaporate the non-conductive polymer matrix which is necessary for the electrospinning process, while conductive polymers have often relatively low molecular weights and are hard to dissolve in common solvents, both factors impeding spinning them solely and making a spinning agent necessary. On the other hand, conductive coatings may disturb the desired porous structure and possibly cause problems with biocompatibility or other necessary properties of the original nanofiber mats. Here we give an overview of the most recent developments in the growing field of conductive electrospun nanofiber mats, based on electrospinning blends of spinning agents with conductive polymers or nanoparticles, alternatively applying conductive coatings, and the possible applications of such conductive electrospun nanofiber mats.

## 1. Introduction

Electrospinning is a relatively simple method to produce nanofibers from diverse polymers or polymer blends [1,2]. Embedding nanoparticles to modify their physical or chemical properties is often reported in the literature [3,4,5]. These composite fibers can in many cases be calcinated afterward to create pure metallic, semiconducting, or other non-polymeric nanofibers [6,7,8].

Generally, electrospinning is performed by pressing a polymer solution or a melt through a needle [9,10] or by coating wires, cylinders, and other objects by the polymer melt or solution [11,12,13,14]. In both cases, a strong electric field is generated by a high voltage which drags the polymer to a substrate, in this way stretching and thinning the polymer drops to create long, fine nanofibers. This means, however, that while most polymers and other materials can unambiguously be embedded in a spinning agent like polyacrylonitrile (PAN) or polyethylene oxide (PEO) [15,16], electrospinning becomes dangerous or even impossible if conductive solutions or melts are used which may form undesired connections between both high voltage electrodes along which the high voltage may discharge, resulting in flashovers.

**Figure 1:**
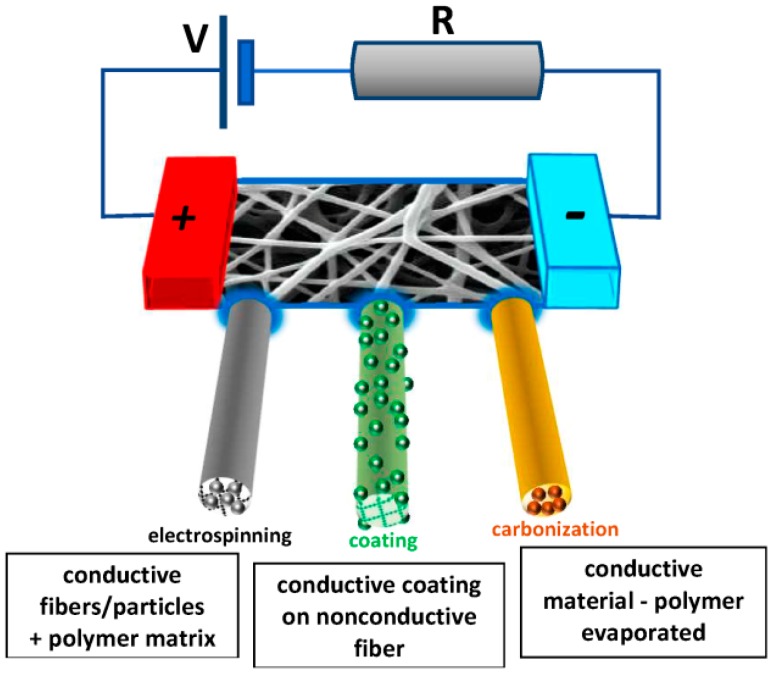
Common techniques to create conductive nanofibers, described in chapters 2–4.

One of the possibilities to nevertheless prepare conductive nanofiber mats is based on the carbonization of polyacrylonitrile, the most often used precursor for carbon nanofibers [17], lignin [18] or other nanofiber mats. These approaches are not discussed in detail here. Instead, we give an overview of different electrospinning techniques that can be applied in the case of conductive polymers as well as diverse after-treatment steps, enabling the preparation of originally non-conductive nanofiber mats which are made conductive afterward (Figure 1), followed by possible applications of such conductive nanofiber mats.

**Figure 2:**
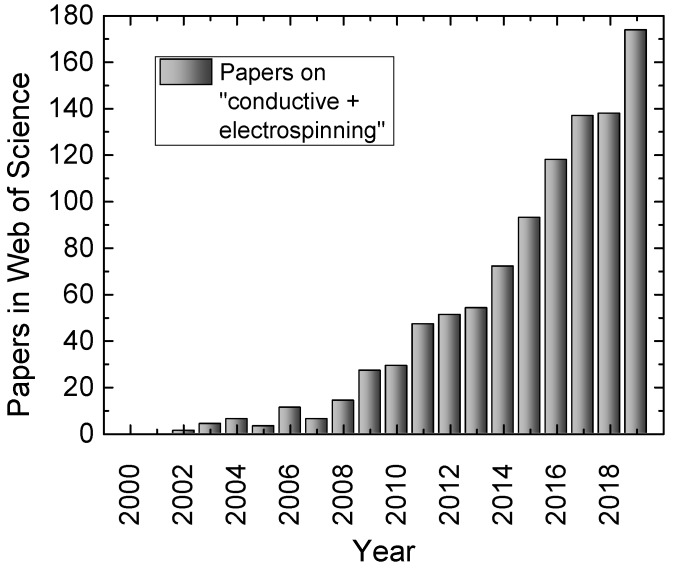
Number of papers on “conductive” and “electrospinning”, listed in the Web of Science (analyzed on 23 December 2019).

The number of studies on electrospun conductive nanofiber mats has strongly increased during recent years due to their large field of applications, as visible in Figure 2. It must be mentioned, however, that due to the broad spectrum of applications, “conductive” nanofibers do not always have conductivities of comparable orders of magnitude in these studies, but also span a wide range of conductivities or sheet resistances, respectively.

## 2. Electrospinning from Conductive Solutions or Melts

One possibility to create conductive nanofibers by electrospinning is based on including conducting nanoparticles or nano-sheets and sintering after electrospinning to remove the non-conductive polymeric matrix (Figure 2). Li et al. describe the process of creating a hydrophobic and conductive composite nanofiber mat in this way. In their needle-based electrospinning setup, two needles on a positive voltage share a common cylinder substrate on negative voltage. By co-electrospinning trimethylethoxysilane (MTES) and a conductive polyvinylpyrrolidone/graphene solution, they removed the PVP by sintering at 500 °C. The resulting MTES/graphene nanofiber mats showed sheet resistance up to nearly 2000 S/m [19].

Wang et al. prepared conductive core-shell nanofiber mats by needle-based electrospinning a solution from multi-wall carbon nanotubes (CNTs) in polycaprolactone (PCL) and silk fibroin. While the conductivity of these nanofiber mats is not reported in their paper, the nanofiber mats were found to allow for neurite extension and cell migration along the nanofibers [20].

**Figure 3:**
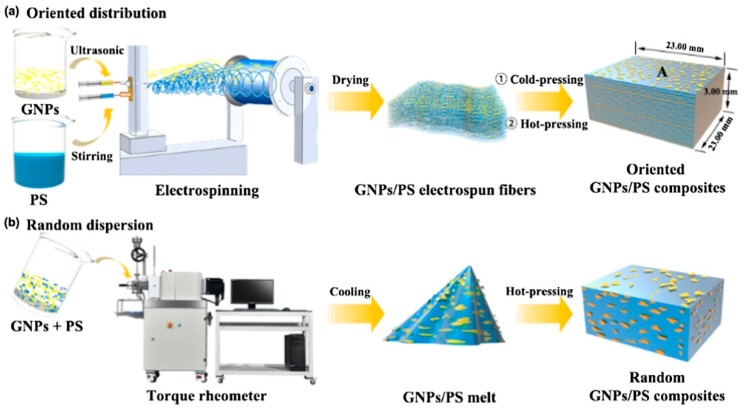
Comparing the oriented distribution of graphite nanoplatelets in composites with polystyrene, prepared by electrospinning, with random dispersion. Reprinted from [23], with permission from Elsevier.

Generally, however, embedding such special carbon modifications does not necessarily result in very high conductivities. With functionalized single-wall carbon nanotubes (CNTs), a conductivity of 1 S/m was reached [21], while even high amounts of multi-wall CNTs resulted in only 10 nS/cm [22]. Using graphite nanoplatelets as fillers in electrospun polystyrene nanofibers which were cold- and hot-pressed after spinning, Guo et al. reached a higher value of approximately 1 S/cm for the highest graphite loading, as depicted in Figure 3 [23]. Shrestha et al. included functionalized multi-wall CNTs in a polyurethane/silk spinning solution and found conductivities of nearly 60 µS/cm for the resulting nanofiber mats, as compared to values below 1 µS/cm for pure polyurethane (PU) or PU/silk nanofiber mats [24]. Combining electrospraying of polyurethane (PU) with simultaneous electrospraying of multi-wall CNTs, Shokraei et al. reached conductivities between 10^−5^ and 10^−2^ S/cm, as compared to the conductivity of the pure isolating PU of 10^−10^ S/cm [25].

Abedi et al. report on conductive chitosan/PEDOT:PSS nanofiber mats [26]. The complicated electrospinning process of chitosan solutions is often attributed to their low conductivity [27]; a problem that can be solved by using different spinning agents [28,29]. They showed that adding up to 1% PEDOT:PSS was sufficient to increase the conductivity of the resulting nanofiber mat by two orders of magnitude, in this way significantly increasing cell proliferation in cardiac tissue engineering.

Adding conductive MoS_2_ nano-sheets to a nonconductive nylon spinning solution resulted in s significantly increased conductivity of the solution of approximately 20 µS/cm; however, this value is still far below a critical value for electrospinning [30]. Nevertheless, the resulting increase of conductivity of the nanofiber mats was sufficient to increase cellular attachment and cell proliferation, and even to induce cardiogenic differentiation of mouse embryonic cardiac cells.

Conductive polymers are usually hard to electrospin solely because they often show a low solubility in most solvents and usually have a low molecular weight which impedes fiber formation. Blending them with spinning agents is one possibility to prepare conductive nanofiber mats from such conductive polymers [31]. Bittencourt et al., e.g., used electrospinning from different PVA/polyaniline (PAni) solutions with de-doped PAni, resulting in nanofiber mats with conductivities around 35 nS/m which was sufficient for the use as ammonia gas sensor [32].

Akcoren et al. prepared nanofiber mats from blends of polypyrrole and poly(butyl acrylate-co-methyl methacrylate), in this way, increasing the alternating current (AC) conductivity to a range of 0.4–0.5 µS/cm [33]. Poly(caprolactone)(PCL)/PAni nanofibers were electrospun using the camphorsulfonic acid doped green form of PAni by Garrudo et al., resulting in much higher conductivities in the range of 10^−4^–10^−1^ S/cm [34]. Liu et al. used a side-by-side spinneret to spin camphoric acid doped PAni together with PEO and found an increased spinnability combined with conductivities between 10^−6^ and 10^−4^ S/cm [35].

**Figure 4:**
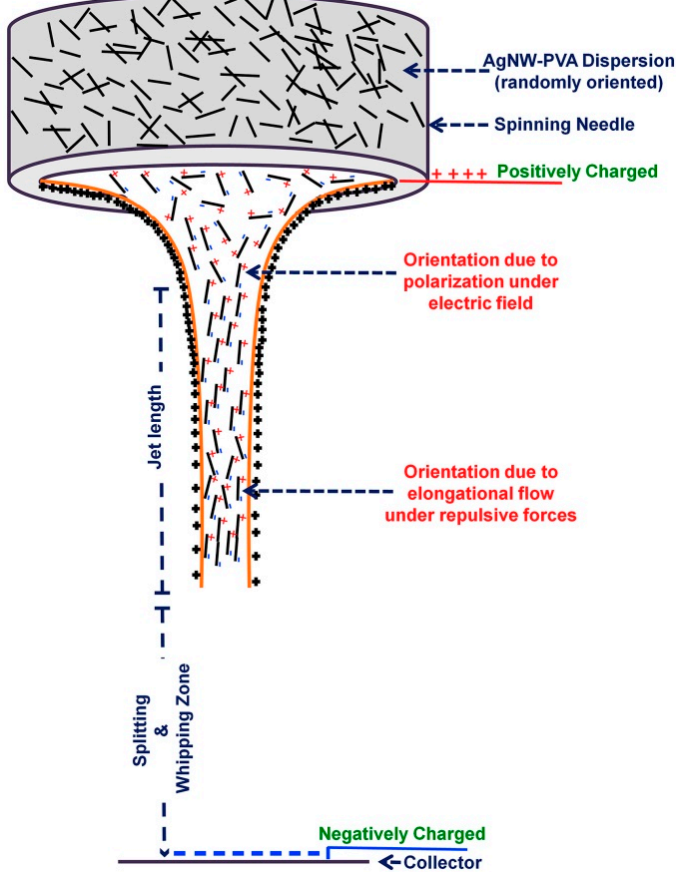
Alignment of silver nanowires inside nanofibers during the electrospinning process. Reprinted from [36], with permission from Elsevier.

These conductivities, however, are still low as compared to values that can be achieved by adding conductive nanowires, etc. Yadav et al. recently reported on polyvinyl alcohol (PVA) electrospun nanofiber mats which included approximately 1/3 weight percent of silver nanowires [36]. They found that this approach resulted in conductivity of more than 650 S/cm, i.e., a much higher value than reached by the aforementioned conductive polymer blends. This high conductivity was attributed to the orientation in the jet during electrospinning, as depicted in Figure 4.

## 3. Electrospinning and Subsequent Calcination of a Polymer

**Figure 5:**
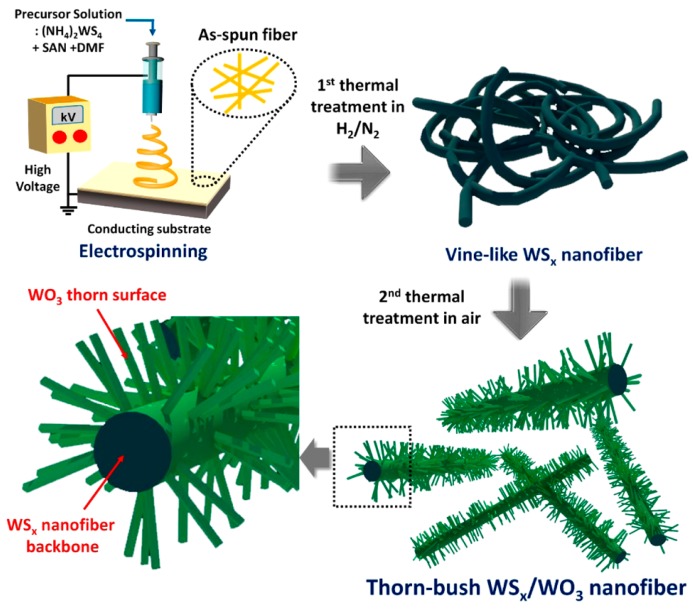
Electrospinning (NH_4_)_2_WS_4_/styrene acrylonitrile and double thermal treatment to prepare WS_x_/WO_3_ thorn-bush nanofibers. Reprinted with permission from [39]. Copyright (2016) American Chemical Society.

Completely metallic nanofibers were produced by electrospinning copper(II) acetate and PVA in a needle-based setup with a rotating collector, followed by calcination (Figure 2) to maintain pure CuO nanofibers which were used as translucent conductive layers with sheet resistances around 0.4–5.4 MΩ [37]. Silver-electroplated electrospun nickel microfibers showed much lower sheet resistance of less than 0.2 Ω [38]. For the application as anode materials in Na-ion batteries, Ryu et al. prepared hierarchically structured WS_x_/WO_3_ thorn-bush nanofibers by electrospinning (NH_4_)_2_WS_4_/styrene acrylonitrile solutions in dimethylformamide (DMF) and two subsequent thermal treatments, resulting in the growth of thorns vertical to the nanofiber surface which further increased the fiber surface [39] (Figure 5).

## 4. Conductive Coatings

**Figure 6:**
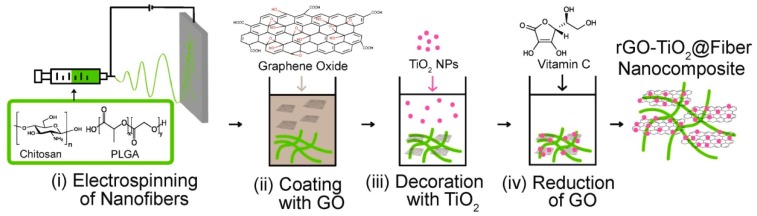
Preparation of reduced graphene oxide/TiO_2_ enabled nanofibers. Reprinted from [40], with permission from Elsevier.

Another possibility to create conductive nanofibers, also based on a two-step process, is performed by applying a conductive coating on non-conductive or weakly-conductive nanofibers (Figure 2). Fausey et al., e.g., prepared a nanofiber mat from a chitosan/poly(lactic-co-glycolic) acid polymer blend by needle-based electrospinning and afterward dip-coated this substrate by conductive graphene oxide, followed by dip-coating in TiO_2_ and afterward reduction of the graphene oxide with vitamin C to increase its conductivity (Figure 6), resulting in increased arsenic oxidation due to the faster shuttling of electrons from the valence band of the TiO_2_ and thus reduction of electron-hole recombination [40].

Similarly, Ahmed et al. prepared a poly(vinylidene fluoride-co-trifluoro ethylene) (PVDF-TrFE) nanofiber mat by electrospinning and spray-coated it with a mixture of multi-wall CNTs and reduced graphene oxide five times, before this nanofiber mat was finally coated with PEDOT. This resulted in a conductivity of nearly 4000 S/cm, depending on the exact material combination, and allowed for using the nanofiber mat as a conductive electrode in a piezoelectric pressure sensor [41]. Conductivities of approximately 0.3 S/m were found by Li et al. who coated electrospun PAN yarn with multi-wall CNTs [42].

Polypyrrole (PPy) is often used for the preparation of artificial muscles. Ebadi et al. produced polyurethane (PU) nanofibers by needle-based electrospinning and afterward coated PPy onto these PU nanofibers. For this, pyrrole monomer with LiTFSI was dissolved in water, and an oxidizing agent was gradually added to polymerize PPY on the PU nanofibers, a process in which bonding occurred by radical cations [43]. Similarly, the same group coated PU nanofibers with a p-toluenesulfonate doped PPy layer, resulting in electrical conductivity of approximately 276 S/cm. These nanofiber mats could be used as bending actuators [44]. To prepare scaffolds for neural cell growth, Xu et al. prepared poly(l-lactide acid)-PCL fibers and coated them electrochemically with chitosan and PPy, resulting in conductivities of approximately 1 S/m [45]. In-situ polymerization of pyrrole on Fe_3_O_4_/polylactic acid-glycolic acid resulted in magnetic nanofibers with a conductivity of up to 0.58 S/cm [46].

Dognani et al. coated an electrospun polyvinylidenefluoride-co-hexafluoropropilene (VDF-HFP) nanofiber mat with PAni, resulting in modified pore sizes, and water contact angles from the clearly hydrophobic surface of pure PVDF-HFP nanofiber mats to hydrophilic surfaces of PVDF-HFP/PAni nanofiber mats [47]. Pure PEDOT nanofibers were prepared by Laforgue and Robitaille by applying an EDOT coating on an electrospun PVP nanofiber mat, polymerizing it to PEDOT and afterward calcinating the PVP core, in this way creating nanofibers with a conductivity of approximately 60 S/cm [48]. Similarly, pure PAni nanofibers were prepared by coaxial electrospinning of a PAni shell around a poly(methyl methacrylate (PMMA) core, resulting in nearly identical conductivities [49], while PVDF nanofibers with an aniline coating polymerized on them resulted in one order of magnitude lower conductivity [50].

Coating PU nanofibers with silver nanowires, Kim et al. found sheet resistances between 0.7 Ω and 510 Ω, depending on the areal weight of the coating [51].

## 5. Applications of Conductive Electrospun Nanofiber Mats

### 5.1. Electromagnetic Shielding

**Figure 7:**
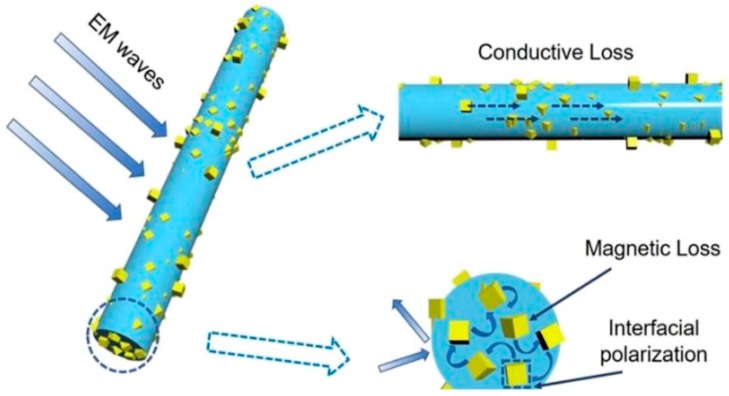
Electromagnetic wave absorption due to magnetic and dielectric losses. Reprinted from [53], with permission from Elsevier.

One of the large areas in which electrospun nanofiber mats are used is electromagnetic shielding. Typically, lightweight electromagnetic (EM) wave absorbers are prepared as heterogeneous structures from magnetic and dielectric loss materials, with the heterogeneous structure supporting the interaction between an electromagnetic wave and absorber [52]. This results in a strong use of combinations of magnetic loss materials like magnetic metals with dielectric loss materials like carbon in different modifications for the preparation of lightweight EM composite absorbers, as depicted in Figure 7 [53,54,55,56]. Nanofiber mats electrospun from other combinations such as ZnO/C are also reported to show good microwave absorption [57].

### 5.2. Energy Storage

Another application of conductive nanofiber mats are electrodes of lithium-ion batteries. Here again, metallic and carbon-based materials are often combined to gain a sufficient conductivity. Typically, the anode is prepared from MgFe_2_O_4_ in combination with graphene [58], carbon nanotubes [59] or graphene aerogel [60,61]. MoS_2_/carbon nanofiber membranes were prepared by needle-based electrospinning and carbonization of the PAN-based precursor and used as binder-free anodes for sodium-ion batteries [62].

**Figure 8:**
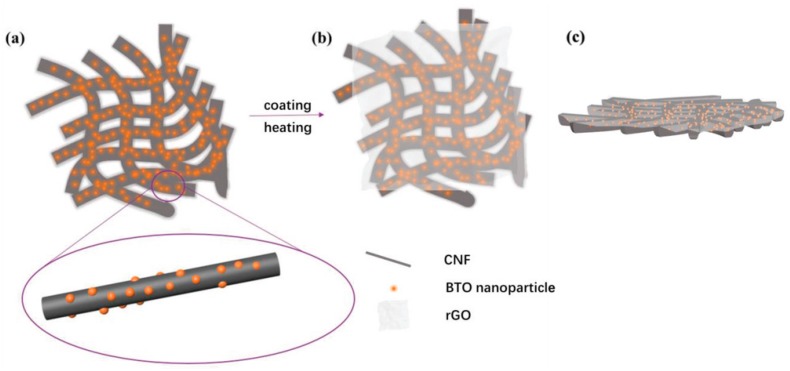
Pure BaTiO_3_@CNF interlayer and interlayer loaded with rGO. Reprinted from [63], with permission from WILEY-VCH Verlag GmbH & Co. KGaA. (**a**) BaTiO_3_@CNF interlayer; (**b**) rGO/BaTiO_3_@CNF interlayer in top view; (**c**) rGO/BaTiO_3_@CNF interlayer in side view.

Interlayers for Li-S batteries were prepared by Zhang et al., combining a reduced graphene oxide layer with BaTiO_3_ decorated carbon nanofibers prepared by electrospinning and subsequent calcination (Figure 8), resulting in low resistances around 30 Ω in the fresh state and around 6 Ω after cycling, resulting in a high rate performance and cycling performance [63].

Supercapacitors, on the other hand, can be created by firstly electrospinning TiO_2_ nanofibers from a solution of Ti(OC_4_O_9_)_4_ and poly(vinyl pyrrolidone) (PVP), followed by calcination to remove the polymer and retain the pure semiconductive nanofibers. Next, nitridization via ammonia annealing resulted in highly conductive TiN nanofibers. These nanofibers were afterward coated with MnO_2_ nanosheets, resulting in increased specific capacitance and cycle stability [64].

### 5.3. Electronic Components

Even memristors were produced by conductive nanofiber mats. Lapkin et al. used electrospinning to produce polyamide-6 nanofiber mats on which PAni was polymerized, resulting in a conductivity around 1 S/cm. Combined with a solid polymer electrolyte and a silver counter electrode, a memristor could be realized which showed resistive switching due to a voltage-controlled change in the PAni redox state [65]. Döpke et al. suggested producing conductive magnetic nanofiber mats for data storage and transfer [4].

### 5.4. Tissue Engineering and Cell Growth

Tissue engineering generally is often based on electrospun nanofiber mats. In order to engineer cardiac tissue, it is not only necessary to create porous nanofiber scaffolds, but these scaffolds should also mimic the extra-cellular matrix of the target tissue, i.e., should be conductive in case of growing cardiac muscle tissue on them with undisturbed intracellular signaling [66,67]. In general, scaffolds with embedded conductive materials often show advances against non-conductive nanofiber mats, whether prepared with PAni, PPy or CNTs [68,69,70].

Nekouian et al. report on conductive electrospun nanofiber mats, prepared from PCL/PPy/multi-wall CNTs which were used to examine the influence of electrical stimulation on the photoreceptor differentiation of mesenchymal stem cells, showing that rhodopsin and peripherin gene expressions could significantly be increased by the electrical stimulation [71]. Rahmani et al. used silk fibroin nanofibers filled with conductive reduced graphene oxide, resulting in electrochemical series resistances around 20–30 Ω, to grow conjunctiva mesenchymal stem cells under electrical stimulation and found formation of neuron-like cell morphology and alignment along the electrical field [72]. PCL/PAni scaffolds with conductivities up to approximately 80 µS/cm were used by Garrudo et al. for the cultivation of neural stem cells, showing that the typical cell morphology was retained, and the nanofiber mats were biocompatible [34]. Even lower values of approximately 1 µS/cm were reported by Ghasemi et al. who doped electrospun polyethylene terephthalate (PET) nanofibers with graphene oxide to prepare cardiac patches for cardiac regeneration after myocardial infarcts [73]. For the same purpose, Walker et al. suggested using electrospun gelatin methacryloyl with bio-ionic liquid to combine adhesive and conductive properties [74].

**Figure 9:**
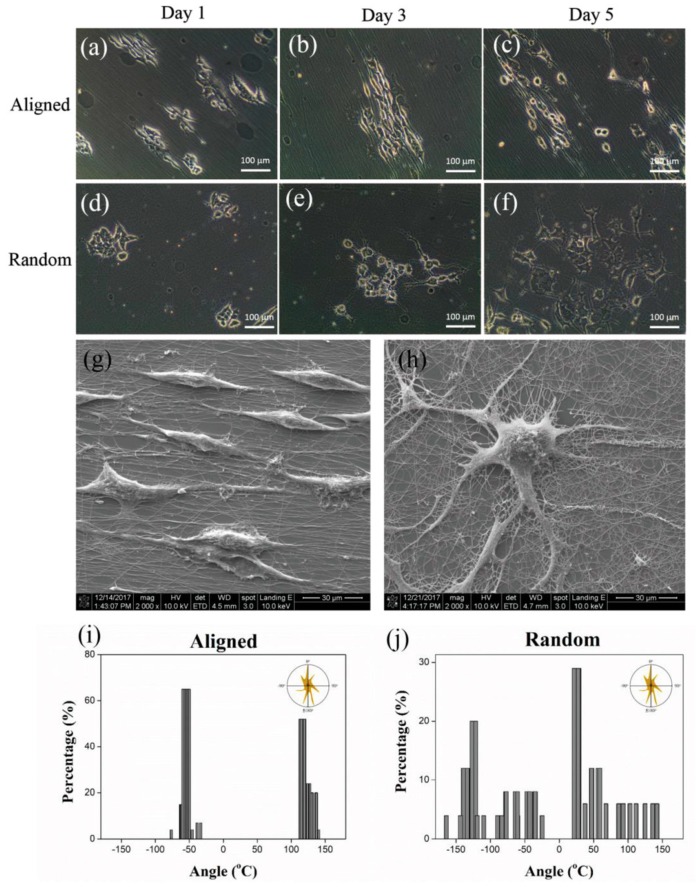
PC12 cells grown on aligned (**a**–**c**,**g**) and random nanofibers (**d**–**f**,**h**), resulting in oriented or random neurites (**i**,**j**). Reprinted from [78], with permission from WILEY-VCH Verlag GmbH & Co. KGaA.

Cell proliferation and gene expression could also be optimized by doping PAni scaffolds with graphene oxide and plasma treatment to hydrophilize the fiber surface [75]. Attachment, spreading and proliferation of fibroblasts and endothelial cells was optimized by tailoring the concentration of multilayer graphene flakes in electrospun polyurethane nanofiber mats [76]. Embedding reduced graphene oxide in electrospun poly(ester amide) (PEA) and PEA/chitosan scaffolds increased cardiac differentiation [77]. Similarly, electrospinning PEO/PEDOT:PSS nanofibers showed a positive effect on neurite outgrowth, i.e., neural differentiation of neuron-like model cells, which is especially interesting since a spin-coated PEO/PEDOT:PSS film showed contact repulsion limiting cell attachment and proliferation (Figure 9) [78]. 

Osteoblast cells were found to grow and proliferate well on electrospun poly(l-lactic acid)/PAni/p-toluene sulfonic acid nanofiber mats [79]. Keratinocytes were shown to grow on electrospun PAN/PPy and PAN/PPy/CNT nanofiber mats [80]. Coating electrospun polyurethane nanofibers with PAni reduced the water contact angle significantly, resulted in a certain anticoagulant effect and was found supportive for cell adhesion, proliferation, and extension [81].

### 5.5. Dye-Sensitized Solar Cells

**Figure 10:**
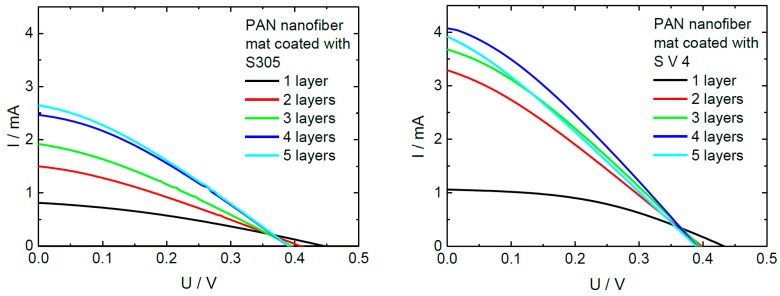
Current-voltage curves of DSSCs, prepared with different PEDOT:PSS counter electrodes. Reprinted from [82], originally published under a CC BY license.

Counter electrodes of dye-sensitized solar cells (DSSCs) were prepared by coating an electrospun nanofiber mat with PEDOT:PSS. Juhász Junger et al. used several dip-coating steps to optimize the electrode conductivity while partly retaining the nanostructured surface and thus the large contact area with the neighboring layers (Figure 10) [82]. The optimum number of layers resulted in a sheet resistance around 150 Ω, reduced from approximately 550 Ω for a single coating layer [82,83]. A similar approach was recently suggested by Kohn et al. who prepared fully electrospun DSSCs with both electrodes prepared by separately dip-coating them in PEDOT:PSS [84]. 

Eslah and Nouri, on the other hand, used spin-coating of WO_3_ nanoparticles on electrospun PAN/PAni nanofibers to prepare counter electrodes of DSSCs [85]. For the possible use in LEDs and solar cells, Jiang et al. developed transparent conductive electrodes by electrospinning copper nanofibers and immersing them in silver ink as a protective layer, resulting in sheet resistances below 10 Ω [86].

### 5.6. Hydrogen Evolution

**Figure 11:**
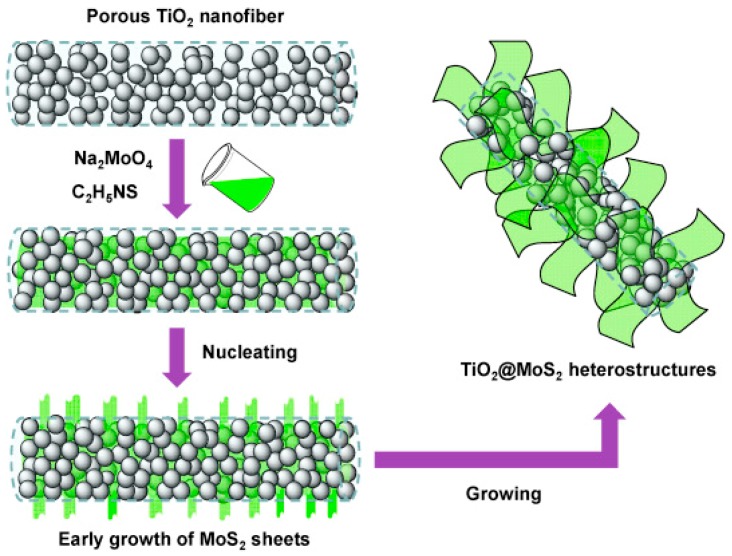
Nucleation and growth of MoS_2_ nanosheets on porous TiO_2_ nanofibers. Reprinted from [91], with permission from Elsevier.

Another interesting application is hydrogen evolution. Sun et al. most recently prepared electrospun carbon/Ni/Mo_2_C nanofibers which were used as electrocatalysts in hydrogen evolution reaction in an alkaline electrolyte [87]. Li et al. used nitrogen-doped carbon/Ni nanofibers decorated with Pt for hydrogen evolution, resulting in a high electrochemical activity combined with reduced usage of Pt [88]. Zhang et al. prepared binder-free MoS_2_/carbon nanofiber electrodes by electrospinning and carbonization of the resulting nanofibers, allowing them to tailor the porosity chemically, which could be used for electrocatalytic hydrogen production [89]. Rheem et al. used a hierarchical structure of MoS_2_ nanosheets on conductive MoO_2_ nanofibers, gained by electrospinning, calcination, and sulfurization, to increase the hydrogen evolution reaction [90]. A similar hierarchical structure was prepared earlier by Liu et al. who used porous electrospun TiO_2_ nanofibers as a substrate for growing MoS_2_ nanosheets perpendicular to the nanofiber surfaces, resulting in high photocatalytic hydrogen production (Figure 11) [91].

### 5.7. Sensors

**Figure 12:**
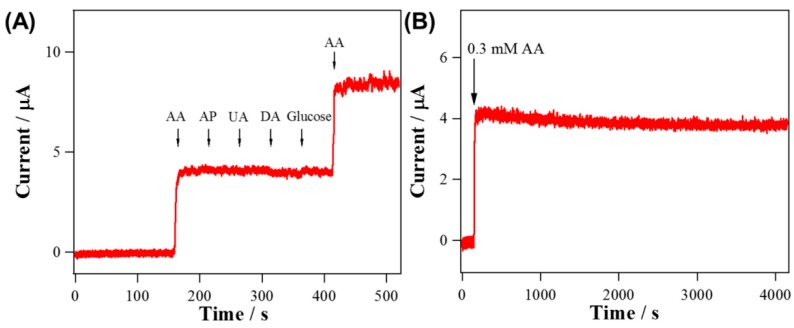
The amperometric response of the WO_3_ nanofiber with RuO_2_ nanorods, showing the stability for detection of L-ascorbic acid (AA) against additions of diverse chemicals (**A**) and against time (**B**). Reprinted from [92], originally published under a CC BY license.

Lee et al. used electrospun WO_3_ nanofibers coated with RuO_2_ nanorods as a sensor for H_2_O_2_ and L-ascorbic acid. They could show that by the addition of the RuO_2_ nanorods, the electrocatalytic activity was increased, and the sensing abilities were significantly improved in comparison with pure WO_3_ nanofibers, as shown in Figure 12 [92].

To sense dopamine, Ozoemena et al. used electrospun PAN/onion-like carbon nanofibers and found a high conductivity and sensitivity of the resulting nanofibers [93]. By electrospinning polystyrene/polyhydroxibutyrate filled with graphitized carbon and partly doped with porphyrin on an interdigitated electrode, Avossa et al. prepared gas sensors for volatile organic compounds [94].

Shaker et al. developed a polyurethane/PEDOT:PSS electrospun nanofiber mat which exhibited a resistance of approximately 3 kΩ and could be used as a reliable strain gauge sensor [95]. Yang et al. coated highly conductive MXene sheets on electrospun PU nanofibers mats to produce highly sensitive strain sensors [96]. Flexible strain sensors with up to 1000% elongation were prepared from conductively coated electrospun styrenebutadiene-styrene copolymer [97]. A similar stretchability was reached by Ren et al., electrospinning a thermoplastic polyurethane nanofiber mat with a wavelike structure, followed by wrapping CNTs around the nanofibers [98]. Wrapping conductive nanofiber yarn produced from graphene oxide-doped PAN nanofibers with in-situ polymerized PPy around elastic yarns results in high sensitivity and repeatability, in this way enabling detection or breathing or human motion [99].

Harjo et al. developed conductive fiber scaffolds by coating electrospun glucose-gelatin nanofiber mats with polypyrrole and investigated their electro-chemo-mechanical response, showing stable actuation for more than 100 cycles as well as reasonable sensor properties [100]. They found conductivities of approximately 3 µS/cm in the unstretched state and approximately half this value when stretched in aqueous or organic electrolyte solutions.

Finally, Table 1 gives an overview of the conductivities mentioned in this article, reached with different methods, again showing the broad range of conductivities reached by different methods and sufficient for various applications.

## 6. Conclusions

In this review, we report we give an overview of the most recent developments in the research area of conductive electrospun nanofiber mats. As well as the possible applications, varying from biomedicine to sensors to batteries to hydrogen evolution, the range of conductivities achievable with different methods is wide. While conductivities in the range of some 10 µS/cm are sufficient for some biotechnological applications, some techniques such as embedding silver nanowires into the electrospinning solution or coating nanofiber mats with diverse conductive materials result in high conductivities of some 100 to some 1000 S/cm. In many cases, the authors of the cited studies report on additional advantageous findings, such as enhanced biocompatibility or improved fiber diameters.

While this review can only give a short overview of the most recent development, it aims at supporting the growing number of researchers working in this highly interesting field of conductive nanofiber mats to find the best solutions for their own applications.

## Figures and Tables

**Table 1:** Examples of electrical conductivities of nanofiber mats mentioned in this paper, sorted by conductivity.

Nanofiber Materials	Conductivity/(S/cm)	Ref.
PVA/polyaniline	3.5 × 10^−10^	[32]
Multi-wall CNTs in polystyrene	10^−8^	[22]
Polypyrrole/poly(butyl acrylate-co-methyl methacrylate)	5 × 10^−7^	[33]
Polyethylene terephthalate/graphene oxide	10^−6^	[73]
Glucose-gelatin coated with polypyrrole	3 × 10^−6^	[81]
Multi-wall CNTs in a polyurethane/silk	6 × 10^−5^	[24]
PCL/PAni	8 × 10^−5^	[34]
Camphoric acid doped PAni/poly(ethylene oxide)	10^−6^–10^−4^	[35]
PAN coated with multi-wall CNTs	3 × 10^−3^	[42]
Multi-wall CNTs/polyurethane	10^−5^–10^−2^	[25]
Poly(caprolactone)/PAni	10^−4^–10^−1^	[34]
Poly(l-lactide acid) coated with chitosan/ polypyrrole	10^−2^	[45]
Fe_3_O_4_/polylactic acid-glycolic acid coated with pyrrole	0.58	[45]
Graphite in polystyrene	1	[23]
Polyamide-6 nanofiber mats coated with PAni	1	[65]
Trimethylethoxysilane/graphene	20	[19]
PEDOT	60	[48]
PU coated with p-toluenesulfonate doped PPy	276	[44]
Silver nanowires in polyvinyl alcohol	650	[36]
Poly(vinylidene fluoride-co-trifluoro ethylene) coated with multi-wall CNTs and reduced graphene oxide	4000	[41]
